# Patient-derived xenograft models in musculoskeletal malignancies

**DOI:** 10.1186/s12967-018-1487-6

**Published:** 2018-04-23

**Authors:** Wan Lu, Tu Chao, Chen Ruiqi, Su Juan, Li Zhihong

**Affiliations:** Department of Orthopedics, The Second Xiangya Hospital, Central South University, Changsha, 410010 Hunan People’s Republic of China

**Keywords:** Soft tissue sarcoma, Bone neoplasm, Patient-derived xenografts, PDX, Animal model, Precision medicine

## Abstract

Successful oncological drug development for bone and soft tissue sarcoma is grossly stagnating. A major obstacle in this process is the lack of appropriate animal models recapitulating the complexity and heterogeneity of musculoskeletal malignancies, resulting in poor efficiency in translating the findings of basic research to clinical applications. In recent years, patient-derived xenograft (PDX) models generated by directly engrafting patient-derived tumor fragments into immunocompromised mice have recaptured the attention of many researchers due to their properties of retaining the principle histopathology, biological behaviors, and molecular and genetic characteristics of the original tumor, showing promising future in both basic and clinical studies of bone and soft tissue sarcoma. Despite several limitations including low take rate and long take time in PDX generation, deficient immune system and heterologous tumor microenvironment of the host, PDXs offer a more advantageous platform for preclinical drug screening, biomarker identification and clinical therapeutic decision guiding. Here, we provide a timely review of the establishment and applications of PDX models for musculoskeletal malignancies and discuss current challenges and future directions of this approach.

## Background

Musculoskeletal malignancies comprise a group of very rare and heterogeneous malignant tumors with more than 70 subtypes; they arise from cells of mesenchymal origin and often exhibit a highly aggressive biological behavior [1]. Prognostic improvements have been achieved by surgery combined with radiotherapy or neoadjuvant chemotherapy since the 1970s; however, the mortality rate of patients with recurrence and metastasis remains high and shows no signs of improvement [2], highlighting the need for developing new therapeutic approaches.

Although numerous studies have been conducted to identify the underlying tumorigenesis and development mechanisms of sarcoma, leading to the development of new agents, few of them have met the expectations in clinical trials [3], which is also frequently occurred in the drug development of other tumors. The lack of efficacy and safety (both toxicological and clinical) accounts for ~ 60% failures in oncological drug clinical trials [4]. Traditionally, the NCI-60 cancer cell line panel and xenografts derived from it are the most frequently used collection of human malignancy models in vitro and in vivo and have provided valuable information to help us gain better understanding of cancer development [5]. However, it is believed that these long-established tumor cell lines have adapted to the culture environment, which is quite different from the growing conditions of original tumor, through Darwinian selection after serial passages and thus exhibit irreversible alterations in biological properties, including genetic aberrations, loss of specific cell populations, change in growth and invasive patterns, which are all key factors in cancer development and treatment resistance [6, 7]. Given the suboptimal predictive power of cancer cell lines or cell line-derived xenografts (CDXs) in preclinical trials, patient-derived tumor xenografts (PDTXs, PDXs) generated by directly implanting tumor fragments from patients into immunodeficient mice has gained a renewed interest in recent years for their high fidelity of recapitulating tumor biology and heterogeneity in individual patient tumors [8, 9].

The earliest reports of heterotransplantation of musculoskeletal malignancies into nude mice were published around the 1980s [10–12]. Since then, various types of PDXs for musculoskeletal malignancies have been established and used in multiple applications, including drug development, biomarker identification, and guidance clinical decision [13–17]. Some studies have validated that genetic and histopathological characteristics were well preserved between PDXs of soft tissue and bone sarcomas and their parental tumors, based on immunochemistry, flow cytometry, karyotyping, tissue microarray study, RNA and whole-genome sequencing [13, 18–22]. However, the relatively small collections of sarcoma PDX models in contrast to the significant heterogeneity of each sarcoma type restrict the extensive application of PDXs for basic and clinical sarcoma research. In this article, we summarize the methodology for generating bone and soft tissue sarcoma PDX models, provide information of currently available PDX models of musculoskeletal malignancies, list their applications and discuss the challenges and future directions of this approach.

## Generating PDX models of musculoskeletal malignancies

The methodology for generating PDX models of various cancer types, including colorectal, breast, ovarian and cervical cancers has been comprehensively reviewed [23–25]. Most of the PDX models are established by immediately transplanting surgically resected patient tumor fragments into immunodeficient mice. Recent studies also demonstrated that PDX models could be created by using “liquid biopsy” samples, such as circulating tumor cells (CTCs), pleural effusions, and ascites [26]. However, due to the prevalent presence of stem-like, i.e., aggressively growing cells, PDXs generated from liquid biopsies may not reflect the cell types and growth rates of the original tumor. Similar caveats exist for circulating tumor cells and pleural effusions.

**Fig. 1:**
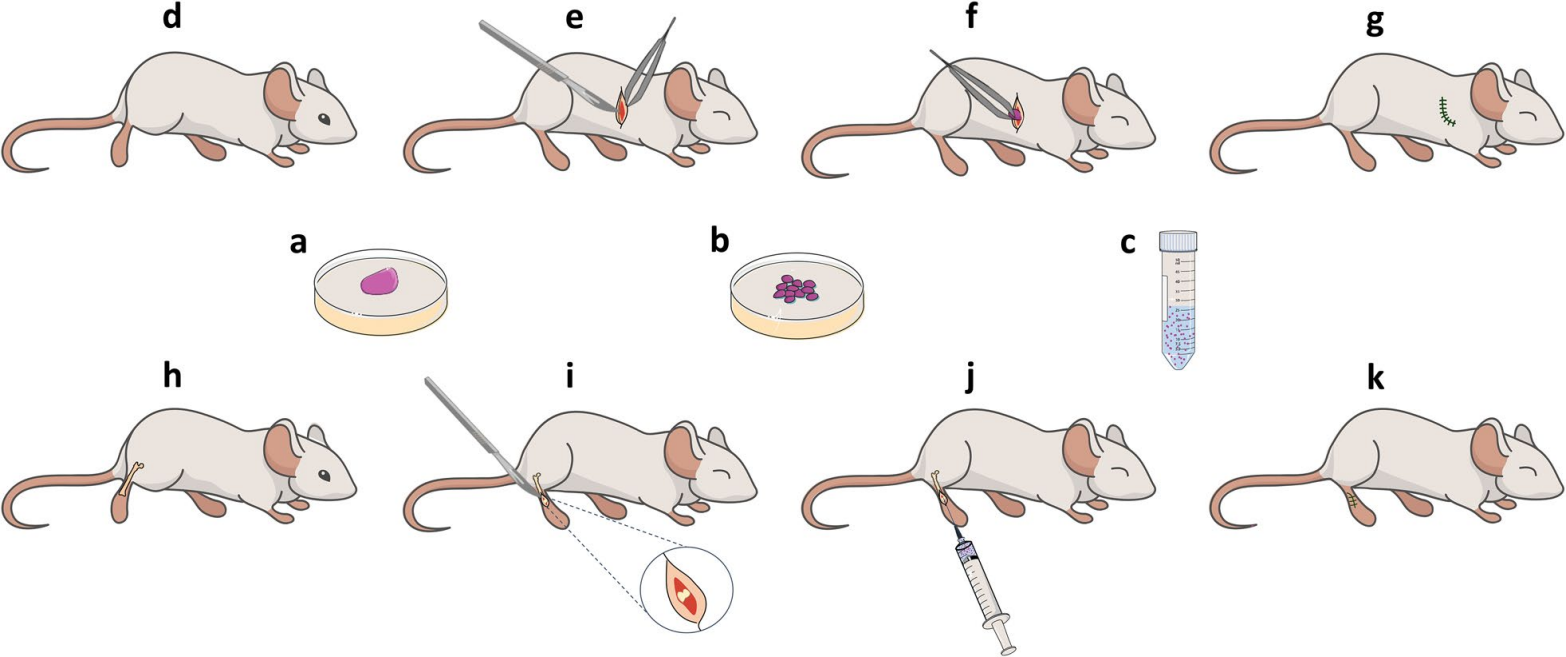
Generation of patient-derived xenografts. **a**–**c** Sample preparation. **a** Patient tumor tissue is collected from surgery resection or biopsy; **b** the tissue is cut or minced into fine fragments in phosphate-buffered saline or medium in a sterile dish, or **c** dissociated in to single cell suspensions; **d**–**g** subcutaneous implantation. **d** A 3–6 weeks old immunocompromised mice is selected as the host; **e** a small skin incision is dissected to discover the subcutaneous space under anesthesia; **f** the tumor fragments are placed subcutaneously with a sterile forceps; **g** suture the skin incision. **h**–**k** Orthotopic implantation. **h** An infant immunocompromised mice is selected as the host; **i** a small skin incision is dissected around the knee joint to discover the femoral condyle under anesthesia; **j** single cell or fine fragments suspension is injected into the femoral shaft using a sterile syringe; **k** suture the skin incision

Generating sarcoma PDX models is the same in essence while differing in minor points. Individual research groups have developed their own specific operating procedures. Briefly, PDX models could be generated by the following steps (Fig. 1).

### Tumour sample collection and processing

After receiving informed consent from the patient, fresh tumor tissues can be collected from biopsies or surgical resection; samples are better preserved in medium with fetal bovine serum (FBS) compared to medium alone and, when possible, keep the tissue at room temperature and do the implantation within 2 h is the best option, if the sample could not be processed immediately, it is better to ship it on wet ice (4 °C) before implantation (< 24 h) [21, 22, 44]. It seems that biopsy samples are not preferred, partly because needle biopsy cannot provide sufficient tumor materials. However, generating PDXs from biopsied tumor samples should be encouraged since it could enable tumor xenografts to be grown from patients who do not receive pretreatment or lose their chance to undergo surgical resection to identify reliable therapies at an earlier stage in the clinical course of the disease. Almost all studies chose to cut or mince tumor samples into fine fragments with a tumor size ranging from 1–8 mm^3^ before xenotransplantation, and only one group used enzymatic digestion to dissociate tumor samples into single-cell suspensions, which was quite convenient for orthotropic implantation [22] (Fig. 1a–c).

### Tumour engraftment

Several studies highlighted that samples were implanted into the mouse within 2 h after collection. In addition, removing necrotic tissue is helpful for successful engraftment. After that, fragments of tumor tissues or single-cell suspensions were implanted into immunocompromised mice either alone or with Matrigel. Utilizing either fine tumor fragments or single-cell suspension has its own merits and defects. Cell-cell interactions within some stromal components could be retained in tumor fragments, thus preserving the original tumor microenvironment, but an artificial tumor selection is barely unavoidable since only a portion of tumors will be implanted. Alternatively, single-cell suspension enables the operator to inject a certain number of homogeneous tumor cells into the host; however, the process of mechanical disruption and enzymatic dissociation may induce cell anoikis, thus hampering cell viability and decreasing engraftment success rate. The addition of Matrigel or other auxiliary components such as estrogen pellets to mimic the complex tumor microenvironment has been reported by several studies with an improved engraftment take rates in PDX models of breast cancer [27, 28], however, suitable supplementation for facilitating the development of PDX models for musculoskeletal malignancies has not yet been described.

Acute transplant rejection is the major reason of tumor engraftment failure. Hence, mouse strains with varying degrees of immune deficiency have been employed for PDX model generation (Table 1). According to the published data, the most frequently used host for establishing primary PDX models of sarcoma is *athymic nude* mouse, which is characterized by the lack of mature and functional T lymphocytes; moreover, the lack of hair on their skin makes nude mice very convenient for observing the growth and response of human tumors to therapies because changes in tumor volume is the most frequently used index in drug efficacy evaluation. *Scid* and *NOD/Scid* (*NS*) mice both harbor severe deficient immune systems featuring the lack of T and B lymphocytes; in addition, *NS* mice have defective innate immunity and are widely used in the transplantation of hematological malignancies. *NOD.Cg*-*Prkdc*^*scid*^*IL2rg*^*tm1Sug*^*/Jic (NOG)* or *NOD/Shi*-*scid IL2Rγ*^*null*^ *(NSG)* mice refer to the most immune deficient mouse strains described to date and carry the *IL2rg*^*null*^ and *Prkdc*^*scid*^ mutations on the *NOD/ShiLtJ* genetic background. *IL2rg*^*null*^ targeted mutation leads to the absence of IL2Rγ, which is essential for the differentiation and function of many hematopoietic cells, thus blocking natural killer (NK) cell differentiation. Meanwhile, the *Prkdc*^*scid*^ mutation results in the dysfunction of the *PRKDC* gene, which encodes a protein responsible for DNA repair in all tissues, including T and B lymphocytes. Thus, it can be inferred that an enhanced therapeutic effect would be achieved when using PDXs established in mice with a *Prkdc*^*scid*^ mutational background to test agents that inhibit DNA damage repair (DDR) or DNA-damaging drugs.

**Table 1:** Characteristics of immunocompromised mouse strains

Mouse strain	Characteristics					Advantages	Applications
	T cells	B cells	NK cells	GNs	DCs		
Nude		±	+	±	+	Low cost Long life span Hairless; easy assessment of tumor volume	Cell line engraftment and patient-derived tumor xenografts Microbiology and immunology research
Scid	−	−	+	+	+	Severe immune deficiency	Cell line engraftment and patient-derived tumor xenografts Infection and immunology research Spontaneous development and metastasis of thymic lymphoma
NS	−	−	±	±	±	Imparied NK cell, DC, and myeloid cell functions Higher engraftment rates of cell lines than in scid or nude mice	Cell line and patient-derived tumor xenografts Infection and immunology research Hematological malignancy research Spontaneous development and metastasis of thymic lymphoma
NSG (NOG)	−	−	−	±	±	Absence of NK cells; the most severely immunodeficient mice High engraftment rates of tumor cell lines and tissues Longer life span than NOD-Scid mice No T/B cell leakage	Widest range of solid and hematologic tumor engraftment Infection and immunology research Humanized model development

“−” absence, “+” normal, “±” impaired, *GNs* -granulocytes, *DC* dentric cells, *NK* natural killer, *NS* NOD-Scid, *Scid* severe combined immunodeficiency, *NOD* nonobese diabetic, *NSG(NOG)* NOD.Cg-Prkdc^scid^IL2rg^tm1Sug^/Jic or NOD/Shi-scid IL-2Rγ^null^

Historically, bone and soft tissue sarcomas were implanted subcutaneously into the flank or dorsal region of mice (Fig. 1d–g), but orthotopic implantation has been developed and applied in several studies in recent years with the aim of better mimicking the initiation and progression the microenvironment of bone neoplasms [22, 29] (Fig. 1h–k). Evidence supports that orthotopic tumors exhibit almost identical genomic profiles as original tumors, while gains or losses of some aberrations are presented in tumors that are subcutaneously generated [29]. Another interesting finding is that spontaneous metastasis could be found in an orthotopic osteosarcoma PDX model but not in subcutaneous models [30]. Igarashi et al. [31] reported their successful experience of establish orthotopic PDX models for RMS. In this research, the authors found that tumor grew faster and could present local recurrence after surgical resection when being implanted into the biceps femoris muscle or quadriceps femoris muscle, rather than under the skin; however, only one patient sample was used in this study. Further large sample size researches are still needed to fully address the most appropriate implantation site of soft tissue sarcoma, in which a PDX model of soft tissue sarcoma can achieve reliable primary tumor growth, stable genomic alteration and both regional and distance metastasis observed in clinical patients.

### Passage of PDX tumors

Serial xenograft passaging is essential for both maintaining and propagating PDX models, as drug testing requires sufficient tumor number for reliable statistical analysis. Passaging of PDX tumors shares the same methodology of primary transplantation. When the tumor diameter of the primary passage PDX reaches 1000 mm^3^, mice could be euthanized, and the tumor harvested. It is important to allow sufficient time for the tumor to grow to a certain volume, as some xenografts may need a long time, up to 6 months, to exhibit obvious growth. Selection of mouse strains could be also be reconsidered, as Stewart et al. [22] chooses *NSG* or *Scid* mice to establish primary passage xenografts and changed to nude mice for passaging, partly aiming to reduce experimental costs; additionally, it seemed that the take rate was not affected by this choice. Moreover, tumor tissue cryopreservation is of great value to create a live bank of early passage tumor cells, which can be done as following steps, (i) cut the tumor tissue into small pieces (e.g. 4 × 2 mm), (ii) place them inside a cryovial containing tissue freezing media (DMSO/FBS, 9:1), (iii) freeze the tissue in a − 80 °C freezer overnight with a freezing container (e.g. Nalgene*Mr. Frosty*Cryo 1 °C) to precisely decrease the temperature at a rate of 1 °C/min, (iv) store it in a liquid nitrogen cryogen tank for long-term preservation. Cryopreserved tissues can be thawed and revived for future research.

### Engraftment success rates

Successful engraftment is defined as reliable tumor growth in primary PDX models with the tumor validated at least by histological analysis. After a comprehensive search of the published reports, we included studies that had a large number of tumor samples and a detailed description of the sample resources to extract data from and summarized the engraftment success rates, tumor sample information and implantation processes in Table 2. In general, variable take rates were observed in the generation of bone sarcoma PDX models, ranging from 24.2% (8/33) to 100% (3/3) and 37.8% (31/82) to 70.9% (22/31) in STS models. Given the significant heterogeneity of the methodology and inconsistent criterion of data reporting, a combined statistical analysis was not performed. Only one study reported higher engraftment rates correlated with tumor stage (primary or recurrent/metastatic) [32], which is contradictory to the observations elsewhere in the literature, showing that metastatic tumors are easier to be successfully engrafted. While tumor histological grade seems to correlate tightly with take rates, most of the PDX models were established from grade III or IV tumors from patients who received pretreatment, such as chemo or radiotherapy, which exerted no effect on tumor growth, however; the specific tumor response to the treatment, which is a rough index of tumor cell viability and thus it may exhibit an inverse correlation with engraftment success rate, was not reported in all of the studies. NSG mice were only used in one large scale study with a moderate take rate of 49% (15/31) in osteosarcoma but a higher take rate of 70.9% (22/31) in STS [22]; however, there was a lack of sufficient data to assess which mouse strain is best suited for generating PDX models of musculoskeletal malignancies.

**Table 2:** Success engraftment rate of bone and soft tissue sarcoma PDXs models

First author/year	Take rate	Take time (weeks)	Host mouse	Tissue source (P/M)	Grade	Previous treatment	Tissue origin (B/SR)	Site (SC/OT)	Tissue size	PDX validation method
Osteosarcoma										
Ishii 1982 [38]	24/30 (80%)	2–12 w	BALB/c nude	NA	NA	NA	NA	SC	0.5 ml fine consistency	Histology
Bauer 1986 [12]	14/25 (56%)	NA	BALB/c nude	NA	III(5)/IV(9)	NA	SR	SC	1–2 mm³	Histology Flow cytometry
Meyer 1990 [14]	8/33 (24.2%)	4 w	CBA/Caj*	P(7)/M(1)	NA	N	SR	SC	2–4 mm³	Histology flow cytometry
Fujisaki 1995 [71]	21/34 (62%)	NA	Nude	NA	NA	NA	SR/Bp	SC	0.5 ml fine consistency	NA
Bruheim 2004 [72]	11/55 (20%)	NA	BALB/c nude	P(7)/M(4)	II(1)/III(2)/IV(8)	Cm(6)/N(5)	SR(6)/Bp(5)	SC	2 × 2 × 2 mm	Histology
Monsma 2012 [45]	3/3 (100%)	NA	Nude	NA	III(3)	NA	SR	SC	Long axis ≤ 3 mm	Histology
Kresse 2012 [21]	9/NA	NA	BALB/c nude	P(7)/M(2)	IV(9)	NA	SR	SC	1–2 mm³	Genomic
Stewart 2017 [22]	15/31 (49%)	NA	NSG	P(8)/M(7)	NA	Cm(9)/N(6)	SR	OT(femur)	1 × 105 cells	Genomic
Ewing sarcoma										
Monsma 2012 [45]	2/3 (67%)	NA	nu/nu nude	NA	NA	NA	SR	SC	Long axis ≤ 3 mm	Histology
Izumchenko 2014 [50]	3/3 (100%)	NA	nu/nu nude	NA	NA	NA	SR	SC	4 mm³	Histology
Stewart 2017 [22]	2/7 (28%)	NA	NSG	P(2)/M(0)	NA	Cm(1)/N(1)	SR	OT(femur)	1 × 105 cells	Histology/genomic
Bone and soft tissue sarcoma										
Hajdu 1981 [32]	37/60 (62%)									
LPS	5/5 (100%)									
LMS	4/9 (44.4%)									
SS	6/6 (100%)									
UPS	6/10 (60%)	NA	Nude	P(18)/M(19)	NA	NA	SR	SC	Fine consistency	Histology
FBS	6/14 (42.8%)									
AGS	3/6 (50%)									
MPN ST	5/6 (83.3%)									
NOS	2/4 (50%)									
Houghton 1982 [11]	6/11 (54.5%)									
RMS	6/11 (54.5%)	NA	CBA/Caj*	NA	NA	NA	SR/Bp	SC	4 mm³	Histology
Boven 1998 [49]	10/21 (48%)	NA	Nude	P(5)/M(5)	NA	NA	SR	SC	2–3 mm diameter	Histology
Hoffmann 1999 [73]	31/82 (37.8%)	NA	Nude	NA	NA	NA	SR	SC	5 × 5 mm	Histology
Monsma 2012 [45]	2/4 (50%)									
RMS	1/3 (33%)									
LMS	1/0 (0)	NA	Nude	NA	NA	NA	SR	SC	Long axis ≤ 3 mm	Histology
SS	1/1 (100%)									
Izumchenko 2014 [50]	18/25 (72%)									
RMS	3/4 (75%)									
LPS	4/5 (80%)									
LMS	2/2 (100%)	6–24 w	nu/nu nude	NA	NA	NA	SR	SC	4 mm³	Histology
SS	1/2 (50%)									
SCS	2/4 (50%)									
NOS	6/8 (75%)									
Stewart 2017 [22]	22/31 (70.9%)									
RMS	14/20 (70%)			P(6)/M(8)		Cm(4)/N(10)				
SS	1/2 (50%)			P(1)/M(0)		Cm(1)/N(1)				
HGS	5/6 (83.3%)	NA	NSG	P(3)/M(2)	NA	Cm(2)/N(3)	SR	OT(femur)	1 × 105 cells	Histology/genomic
EPS	2/3 (67%)			P(1)/M(1)		Cm(0)/N(2)				

*OS* osteosarcoma, *EWS* Ewing sarcoma, *LPS* liposarcoma, *LMS* leiomyosarcoma, *SS* synovial sarcoma, *SCS* spindle cell sarcoma, *UPS* undifferentiated pleomorphic sarcoma, *FBS* fibrosarcoma, *AGS* angiosarcoma, *MPNST* malignant periphery nerve sheath tumor, *NOS* unclassified sarcoma, RMS rhabdomyosarcoma, *HGS* high grade sarcoma, *EPS* epithelioid sarcoma, *SFT* solitary fibrous tumor, * thymectomy and irradiation, *P* primary tumor, *M* metastatic tumor, *SC* subcutaneous, *OT* orthotopic, *SR* surgical resection, Bp biopsy, *Cm* chemotherapy, *N* not received, *NA* not available

## Validation of PDX models for preclinical research

Various type of PDX models for musculoskeletal malignancies [11, 13, 14, 22, 32–38] have been established, with a preference towards osteosarcoma (OS) and rhabdomyosarcoma (RMS), partly due to their relatively higher morbidity. Reviews of several other tumors concluded that PDX models have strong predictive power in preclinical research based on the findings that genetic and histopathological characteristic are well preserved in PDXs [23, 24, 39]. However, it is not known whether the outcome is the same within PDX models of bone and soft tissue sarcoma.

Inconsistent results were found in tissue architecture and cellular morphology in histologic analysis of PDX models and their parental tumors. A large-scale hematoxylin and eosin (H&E) and immunohistochemistry analysis of 39 bone and soft tissue sarcomas revealed a 100% concordance of histopathological characteristics between orthotopic PDX (O-PDX) models and their corresponding tumors [22]. Minor differences were found in several subcutaneous PDX collections (S-PDX). Houghton et al. [11] reported that slightly discrepant tumor necrosis, differentiation and collagen content could be found in 4/6 their HxRh series of rhabdomyosarcoma PDX models. Only a small proportion of osteosarcoma PDXs changed their morphology, such as increased cellularity, dedifferentiation and differentiation, after serial passages [14, 40, 41]. However, some conflicting findings for xenografts apparently deviating from the primary tumor were also revealed. Donhuijsen et al. [42] established twenty-two PDX models of previously untreated soft tissue sarcomas and subjected them to histologic examination and flow cytometry. Significant deviations were observed between the primary PDX model and its original tumor in cell differentiation in 36.3% (8/22) tumors, and some cases even appeared to represent another sarcoma type. In addition, increased frequency of mitosis and reduced connective tissue content were found in serial passages. Similar findings were observed in an osteosarcoma PDX model; Delgado et al. [43] generated three osteosarcoma PDX models using five tumor samples from one patient, but these PDX tumors exhibited two entirely different morphologic subtypes, with one being a firm tumor, while the other a cystic tumor. These differences may be due to genetic instability of xenografts [44]. Moreover, limited tumor samples for engraftment may lead to a loss of some specific cell subpopulations, resulting in an atypical part of the original tumor. The host environment may also be an influencing factor. Given the significant heterogeneity between these studies and the relatively small numbers of each specific sarcoma type, it is difficult to conclude whether PDXs of bone and soft tissue sarcoma can preserve the histopathological characteristics of the original tumor. Thus, a careful histologic analysis of PDXs and their parental tumors is needed before conducting a PDX-based preclinical analysis.

As for the preservation of gene expression profiles, a tight correlation has been observed between early passage bone and soft tissue sarcoma PDX models and their parental patient tumors, with very high Pearson’s correlation coefficients (*r*) ranging from 0.84 to 0.97 in some studies [22, 45]. An artificial neural network (ANN) based on cDNA microarray analysis of a panel of PDX models from the Pediatric Preclinical Testing Program (PPTP) found a relatively low Spearman’s rank-order correlation (r = 0.67, *P *< 0.001) [19]; however, the correlation was still strong. Further research using Affymetrix technology within an increased number of probed genes identified a false discovery rate of < 1.67% in three PDX/parental tumor (osteosarcoma, Ewing sarcoma and rhabdomyosarcoma) comparisons. Gene ontology analysis revealed the biological function of differentially expressed transcripts that were enriched in immune response, cell cycle, RNA metabolism and vesicle-mediated transport, which is consistent with the findings of Monsma et al. [45], suggesting non-randomly altered gene expression profiles in PDX models that may be induced by the loss of normal cells upon transplantation and the logical selective pressure of the immune deficient environment. Moreover, very high Pearson’s correlation coefficients (r = 0.98–0.99) were observed in two osteosarcoma PDX models over 4 generations consistent with the findings of Neale et al. [20]. These reports indicate that PDX models of bone and soft tissue sarcoma can well retain, although not perfectly, the principle gene expression profiles of their original tumors.

Genome-wide analyses have revealed that both clonal composition and copy number variations are reliably preserved in a large collection of PDX models and their parental tumors [20–22, 45]. In a single study of 18 early-passage (initial engraftment or passage 1) and late-passage (passage 4–6) O-PDX pairs, 15/18 pairs exhibited highly similar clonal features [22]. However, although the major chromosomal copy number variations were found to be overlapped, a small proportion of copy number alterations emerged on serial passaging [20, 21]. Additionally, the possible candidate genes were identified to be involved in immune responses, cell cycle and chemoresistance [22], suggesting the presence of significant genomic alterations related to the lack of selection in the new host. Similar findings were also found in PDX models of pancreatic and colorectal cancer [46, 47]. Taking these phenomena into consideration, PDX models of bone and soft tissue sarcoma could be used to test many kinds of therapy strategies that do not depend on an efficient host immune system, and the use of early passage PDX models for preclinical research seems a prudent choice to avoid clonal selection or evolution.

Since alterations in tumor epigenomics and the following gene expression changes greatly influence the initiation and progression of various human malignancies, Guilhamon et al. performed methylome analysis of two osteosarcoma PDX models and discovered an average of 2.0% (n = 9351) of CpG sites displaying major methylation shifts between the primary PDX models and their parental tumors. Moreover, subsequent xenografts were not accompanied by additional changes, as only 0.07% (n = 333) of CpG sites underwent methylation shifts [48]. Although a small number of PDX models were used, this work indicated that osteosarcoma PDX models may be a suitable discovery tool for tumor epigenomics and drug development.

## Clinical correlation of PDX models

A direct comparison of PDX models with that of their corresponding patient tumors regarding response to a specific treatment is essential for evaluating the predictive power in both preclinical research and clinical decision making. Although no large-scale studies have been conducted to clarify this issue, similarities between PDX responses and patient outcomes have been reported in several small cohorts. Boven et al. [49] observed that 4 out of 7 PDX models exhibited identical resistance with that of their parental STS patients to the same chemotherapeutic agents. In another study, 9 out of 10 sarcoma PDX models demonstrated a concordant response with their corresponding patients. In addition, two liposarcoma PDX models from one patient established early during the disease course displayed the same positive clinical response seen in the patient to drugs used during tumor progression, indicating that PDX models also retain therapeutic accuracy over time [50].

## Multiple applications of PDX models

**Fig. 2:**
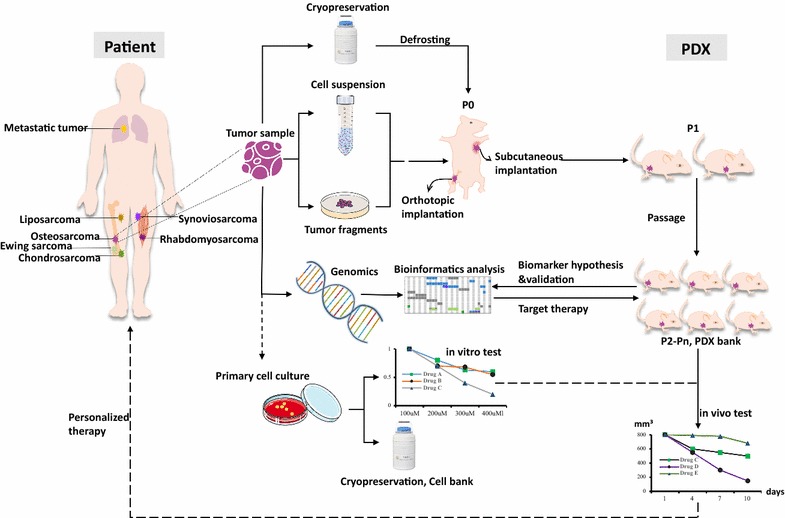
Overall generation and application of patient-derived xenografts in musculoskeletal malignancies. Tumor samples obtained from surgical or biopsy specimens could be separated for three main usage, including generating PDX models, conducting genomic sequencing, and dissociating for primary cell culture. Screening candidate drugs according to doctors’ clinical experiences or with the results of bioinformatics analysis, and (or) in vitro test would provide a reliable personalized therapy strategy for this patient. Moreover, data and PDX model from individual cases could be integrated into a database and use to establish an avatar model bank for future use

Established PDX models of musculoskeletal malignancies have been used in a wide range of research, including drug screening and development, biomarker discovery, clinical treatment guiding and cell-line production (Fig. 2).

### Pre-clinical drug test

Preclinical testing of anticancer agents using in vivo model systems of musculoskeletal malignancies have been performed since the 1980s; however, given the rarity and significant heterogeneity of this group of tumors, this field has received limited attention. Consequently, only a limited number of novel agents have been tested in musculoskeletal malignancies compared to those in other tumor types such as breast and lung cancer (Table 3).

**Table 3:** Selected preclinical studies correlating PDX treatment results with clinical data

Tumor (Refs.)	PDX (n)	Agent	Target	Results	Clinical correlation
OS [33, 54, 56–59, 61]	1	bortezomib	Proteasome	Combination of bortezomib and adriamycin shows strong TGI ablitily	NA
	2	BHQ880	Wnt signaling	Inhibit tumor growth and metasitasis	NA
	15	IFN-α		Significant TGI in all models, dose dependent	NA
	4	IPI-926	Hedgehog signaling	Significant TGI in 2 of 4 models	NA
	1	Pectolinarigenin	STAT3 signaling	Inhibit tumor growth and metasitasis	NA
EWS [60, 66]	2	WNT974	Porcupine	Delay the early metastasis	NA
	1	SN-38 matrices	Topoisomerase I	Delay the tumor recurrence	NA
SS [63, 65, 67]	1	VX970 [63]	ATR	Significant TGI	NA
	3	tazemetostat	EZH2	Significant TGI in 2 of 3 models	NA
	1	ALGP-DOX	Cytotoxic agents	Significant TVI	NA
SFT [33]	2	DOX, IFO, DTIC, eribulin, trabectedin	Cytotoxic agents	DOX/DTIC, DTIC/IFO, DOX/IFO, eribulin, trabectedin shows strong TVI ablitily	Response to DOX/DTIC in PDXs was concordant with clinical data in 6 out of 12 patients
LPS [64, 67]	2	Pazopanib	Tyrosine kinase	Significant TGI	NA
	2	ALGP-DOX	Cytotoxic agents	Tumor volume stabilisation	NA
RMS [50–52, 62]	6	Melphalan	Cytotoxic agents	Produce CR in 5 out of 6 models	10 of 13 untreated patients gain PR after receiving melphalan
	2	Tideglusib	GSK-3β	Negative results	NA
	6	Topotecan, irinotecan	Topoisomerase I	Produce CR in 4 out of 6 models, and CR in 5 out of 6 models, respectively	22 out of 48 patients gain clinical response (CR in 2, PR in 20) after receiving topotecan

*Ref* reference, *n* number, *GSK*-*3 β* glycogen synthase kinase-3beta, *DTIC* dacarbazine, *DOX* doxorubicin, *IFO* ifosfamide, *ADM* adriamycin, *TGI* tumor growth inhibition, *TVI* tumor volume inhibition, *CR* complete regression, *PR* partial regression

Among the several collections of well-characterized PDX models, some have presented promising predictive value for clinical trials. Horowitz et al. [51] used PDX models of RMS to identify a superior oncolytic activity of melphalan than that of frequently used drugs, which was further validated in a phase II clinical trial. The efficacy of protracted schedules of topoisomerase I inhibitors, such as topotecan and irinotecan, was demonstrated in a panel of xenografts and subsequently confirmed in a clinical trial in young patients with advanced RMS [52, 53]. Two patient-derived solitary fibrous tumor xenografts predicted high sensitivity to a combination of doxorubicin and dacarbazine, and a phase II randomized study has been started to validate these preclinical results [33]. PDX models are not only restricted to the testing of cytotoxic agents. For example, early studies of osteosarcoma xenografts indicated biological agents such as interferon-α and antiosteogenic sarcoma monoclonal antibodies resulted in growth arrest in osteosarcoma [54–56]. Recent studies demonstrated that an antagonist of Wnt signaling [57], inhibitors of Hedgehog pathway [58], and proteasomes [59, 60] could slow the growth of osteosarcoma PDX models when used alone or enhance the efficacy of chemotherapy in preclinical studies. Moreover, prolonged metastasis-free survival of patients with Ewing sarcoma and osteosarcoma could be induced by inhibiting the Wnt signaling pathway and STAT3 activation, respectively [61, 62]. A class of new treatments, for example, inhibitors of serine-threonine kinase GSK3β [63] or ATR [64], tyrosine kinase [65], and EZH2 [66], were also validated in PDX models of soft tissue sarcomas. In addition, two novel polymeric drug delivery systems [67, 68] that were capable of delivering SN-38 and doxorubicin directly into the tumor, regardless of the limited aqueous solubility and systematic toxicity, exhibited significant antitumor activity in several bone and soft tissue sarcoma xenografts, showing promising value in local tumor chemotherapy.

### Guiding clinical decision

The concept of individualized care has been proposed for years; however, the rarity of sarcoma and heterogeneity hinder the development of targeted therapies for sarcoma patients. Fortunately, PDX models have shed light on this issue. Stebbing et al. [16] established 22 PDX models of a wide range of sarcoma types to conduct drug testing, and 13 (81%) patients showed a tight correlation between results from their PDX models and clinical outcomes. Notably, 6 patients achieved a significant tumor regression, including one patient who achieved complete tumor regression to the same drug that was effective against their corresponding xenografts. However, 6 patients died before the generation of appropriate models performing drug screening, highlighting the need for technique improvement to shorten the latency between implantation to xenograft establishment.

### Chemo-resistance

Inherently and acquired chemo-resistance occurs in 35–45% of OS patients [69]. The underlying mechanism of chemoresistance has not yet been clearly elucidated, and proposed mechanisms include drug efflux, cell detoxification, and increased repair of DNA damage, apoptosis inhibition and OS stem cells [70]. Thus, better in vivo models are needed to understand the mechanisms of multidrug resistance, identify novel therapeutic strategies to reverse this process and guide clinical decisions of drug administration. In the 1990s, PDX models were not used directly for drug screening but to amplify viable tumor cells for conducting in vitro chemosensitivity analysis, resulting in a relatively lower predictive power (true positive rate, true negative and predictive accuracy were 40, 100 and 66.7%, respectively) [71]. Bruheim et al. [72] established 11 OS PDX models with a take rate of 20% to conduct chemosensitivity analysis of five reference drugs (doxorubicin, cisplatin, methotrexate, ifosfamide and lomustine). Five of these models (TSX pr.2, HPBX, TPX, KPDX, and FTX) were resistant to all compounds tested, suggesting that PDX models can recapitulate multidrug resistance observed in human OS. In addition, PDXs established from patients previously treated with chemotherapy showed a higher resistance rate (80%, 4/5) than PDXs established from patients who did not receive previous chemotherapy (33.3%, 2/6), suggesting that enhanced chemoresistance ability can be induced. A further research using this panel of PDXs to identify potential biomarker of OS chemosensitivity was conducted in 2009. Bruheim et al. [15] mapped the gene expression profiles of 10 PDX (including 8 in their previously report [72]) according to their sensitivity to doxorubicin, cisplatin and ifosfamide. In total, 85 genes for doxorubicin, 74 genes for cisplatin, and 118 genes for ifosfamide were identified. Some of these genes (such as *MAGED*, *HSP27*, *HSP70*, and *MCM2*) were previously reported to be correlated with prognosis or chemotherapy response in osteosarcoma. Furthermore, an enhanced chemosensitivity of OHS cell lines was observed by siRNA-mediated silencing of two of the identified genes (*IER3* and *S100A2*), validating the promising value of PDX to identify biomarker candidates that may be used to predict the chemotherapy response of OS. However, whether or not the treatment responses observed in these OS PDXs are correlated with their tumor of origin have not been clearly elucidated.

In two panels of STS patient-derived xenografts, positive relationship between multidrug resistance proteins (MDR) and doxorubicin sensitivity was observed in the study by Hoffmann et al. [73], while Boven et al. [49] identified a non-significant correlation between the parameters. Instead, low levels of topoisomerase IIα was shown to partly account for chemoresistance of STS. Despite the discrepancies in the results of these two studies, the authors generated a group of well-characterized STS PDX models of different chemosensitivity, which were useful tools for further investigating chemoresistance mechanisms and screening drugs capable of overcoming this long-known problem.

### Mimic spontaneous distal metastasis

A suspected limitation of subcutaneous engraftments of bone and soft tissue sarcomas is that they almost never produce metastasis, which is consistent with the observations from other tumor types [74]. However, a spontaneous metastatic PDX models of OS into the tibia of 31 BALB/c nude mice was generated by Crnalic et al. [30], who injected minced tumor tissues from the 32nd serial passage of a subcutaneously growing human OS PDX model generated from the primary site (femur) of an OS patient with thoracic vertebral metastasis. Lung metastases, as well as some lymph node or liver metastasis, were observed in all of the hosts. Although this PDX was not generated from the initial human tumor tissue but from a serial passage, similar histological characteristics were still retained. More recently, Goldstein et al. [75] and colleagues established a novel spontaneously metastatic model by orthotopically implanting tumor samples into NSG mouse hindlimbs and then subsequently amputated the mouse after sufficient tumor growth to fully mirror the clinical metastasis process of sarcoma patients. Given the lack of metastatic PDX models created from subcutaneous implantation, it can be speculated that injection into the orthotopic site, more specifically the bone environment, is the major reason of metastasis. Thus, this approach may serve as an ideal platform for investigations of the role of the bone environment in regulating tumor invasion and metastasis as well as for the discovery of new drugs against this process.

## Obstacles of PDX development and application

Given the ability of PDX models to recapitulate the primary tumor, it is believed that PDX models will act as an important platform to elucidate new treatments and biomarkers in preclinical research. However, some technical limitations and intrinsic defects of PDX models are still awaiting resolution (Table 4).

**Table 4:** Limitations and future perspectives of PDX models

	Limitations	Future perspectives
Experiment design	No uniform standards in different research groups regarding patient information collection, required mouse strains and model numbers, endpoint selection, positive results definition, and data interpretation	Construct multicenter collaborative network; explore and establish a proper standard
Technical issues	1. Low success rate and high cost of engraftment 2. Long time frame: from engraftment to preclinical test and clinical application 3. Limited assessment tools for monitoring PDX tumor growth and response to therapies	1. Expand tumor sampling method (CTCs); define the best engraftment site (subcutaneous, orthotopic, renal cell capsule) or develop new approach; use PDOs to generate PDXs 2. Explore proper intra- and post- engraftment manipulations 3. Develop noninvasive and cost-effective tools for assessing tumor status
Intrinsic defects	1. Severe immunocompromised host: unsuitable for testing immunotherapy 2. Rapidly stroma substitution: change in the tumor microenvironment; unsuitable for screening agents against stroma elements 3. Dissimilar pharmacokinetics: over- or underestimation of antitumor drug efficacy 4. Tumor selection and evolution: genotype and phenotype alteration across passages	1. Develop immunocompetent models for establishing PDXs: reconstruct human immune system in immunocompromised models; induce immune tolerance to individual tumors in immunocompetent models; use knock-in or novel gene editing technologies generate genetically humanized mice 2. Mimic human tumor environment: inject immortalized human stromal cells 3. Identify the differences between PDX models and humans regrading drug pharmacology 4. Multiple-spot sampling and sample cryopreservation

First, it is very difficult to assess the superiority or inferiority of different methods in establishing PDXs based on current published papers. Among the varying parameters in the generation of PDX models, the factors contributing to a higher engraftment success rates are still unknown. A possible solution is that individual PDX research groups standardize their study reporting, including (i) details of patient information (metastasis, treatment, clinical stage, and histological grade); (ii) sample preservation or transport medium and delay between the isolation of tumors from patients and implantation into mice; and (iii) required tumor volumes and auxiliary components to generate a PDX. Recently, Meehan et al. [76] summarized minimal information standard for a PDX models (PDX-MI), which is a valuable reference for promoting the reproducibility in PDX models and their related studies.

Another drawback of currently used PDX models is the long timeframe between engraftment and generation of sufficient xenografts to conduct drug screening. For primary PDX models, it normally takes more than 4 weeks for a tumor to reach 100 mm^3^ for preclinical study, which is too slow for real-time clinical decision making for high-risk patients. Thus, donors barely benefit directly from their corresponding PDX models. In addition, data interpretation of preclinical studies often differs from that of clinical studies. For example, a drug that slows down the tumor growth compared to the negative control group will lead to a positively significant result; however, if the tumor still progresses in xenografts, this drug does not meet clinical needs.

Mouse strains with different degrees of compromised immune systems have been designed to diminish or avoid immune rejection, thus enhancing PDX engraftment. However, the lack of an intact immune system differs greatly from the living environment of the original tumor, and it impedes the assessment of immunotherapeutic strategies (vaccine, immunomodulators, immunoactivators), such as PD1/PD-L1, CTLA-4 antibody and CAR-T therapies. Meanwhile, the host cannot provide a similar tumor microenvironment as the original tumor since human stroma is gradually substituted by murine components (fibroblasts, blood vessels, immune cells) with the growth and passage of PDXs. Since tumor cells per se cannot recapitulate growth and formation of primary or metastatic lesions and because the initiation and progression of malignant tumors are supported by their microenvironment [77, 78], the loss of stroma of human origin could lead to alterations in the genetic and biological properties of the tumor, thus undermining the practical value of PDXs for preclinical drug testing, as well as hindering the development of novel antitumor agents targeting stromal components.

Tumors are a collection of various cell subpopulations and matrix; however, the tumors samples implanted in mice represent only a “snapshot” of small part of the original tumor. Moreover, these tumors will undergo a natural selection process post-transplantation to adapt to the whole new living environment, and the winner populations are supposed to be the more aggressive cells, which is consistent with the observation that most of the reported PDX models have been generated from histologically high-grade sarcomas. Furthermore, this process would keep on going across passages. Although several large-scale global genetic analysis demonstrated that PDX models exhibit similar genetic properties as their original tumors, some significant aberrations may be lost, and new aberrations may emerge during passaging. However, whether these genetic alterations affect the viability for of PDXs preclinical research should be further clarified in future studies.

## Future perspectives

Based on published reports, the PDXs collections of musculoskeletal malignancies are relatively small, partly due to the rarity of this group of tumors; however, it is more likely because of the limited attention paid in this field. Expanding the PDX library and improving the engraftment success rate of PDXs are of great importance to extend their use for both clinical trials and individualized precision medicine research. Because of the rarity of sarcoma patients and incomparable methodology aspects (sample size needed, preservation media and condition, intra- and post-implantation manipulation, implantation site) among different research groups, constructing an organized multicenter collaboration network, such as the EurOPDX workshop (http://www.europdx.eu) and Patient preclinical testing consortium (PPTC, http://www.ncipptc.org), is an effective way to extend the resource of tumor samples and standardize study design and data reporting. For the long latency in generating PDX models, making clinical decision based on data from previous high-throughput drug screening in large collections of PDX models may be a promising alternative for patients who do not have corresponding PDX models or have highly aggressive malignancies. In addition, combining bioinformatics analysis results to select drug candidates may be an efficient way to narrow down the scope of screening and increase the success rate.

Traditional xenografts of musculoskeletal malignancies were mostly generated by using surgical resection samples; however, generating PDXs from biopsied tumor samples should also be encouraged since it would enable tumor xenografts to be grown from patients who do not receive pretreatment or lose their chance to undergo surgical resection to identify reliable and effective drug therapies at an earlier stage in the clinical course of the disease. Another valuable resource for generating PDX models is circulating tumor cells (CTCs). Several studies have demonstrated that CTCs are strongly correlated with patient sensitivity to therapy and prognosis [79, 80], indicating that CTCs are promising biomarkers for monitoring tumor burden and predicting drug response in high-risk patients. Although technically challenging, CTC-derived PDX models that could also recapitulate the biological properties and drug sensitivity of the primary tumors have been generated from several solid tumors in recent years, including melanoma [81], breast cancer [82], prostate cancer [27], small-cell lung cancer [83] and gastric cancer [84]. Compared to the currently used sample collection methods, CTC sampling is minimally invasive but enables the isolation of tumor specimens that are inaccessible to surgical operation or needle biopsies. More importantly, CTCs allow us to generate PDX models from tumors at any time in the clinical course to adapt our clinical decision and analyze the underlying mechanisms of tumor evolution or dissemination.

Gradual substitution of stroma over time seems unavoidable in PDX models, and it seems that there are no solutions for this issue. A meaningful reference is the work done by Kuperwasser et al. [85], who generated humanized mammary fat pads by injecting irradiated immortalized fibroblasts into NSG mouse mammary fat pads. In light of this research, we can speculate that co-engraftment of immortalized mesenchymal stem cells or cancer-associated fibroblasts with tumor tissues may reconstitute human tumor stroma in PDX models; however, future studies are warranted.

**Fig. 3:**
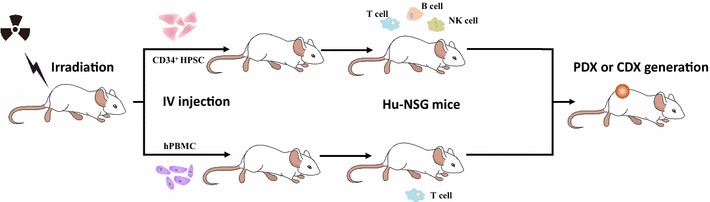
Overall generation of human immune systems in immunocompromised mice. After subjecting to irradiation, purified CD34^+^ HPSCs or hPBMCs are injected into the peripheral blood of NSG mice to generate humanized mice for CDX or PDX generation

Immunocompromised mouse is currently the standard host for creating PDXs. As mentioned above, the lack of an intact immune system may alter the genetic profiles of PDX models and restrict them from being employed for immunotherapy development. Can we establish PDX model in immunocompetent mice or reconstituted human immune systems in immunocompromised mice? Several studies have demonstrated this feasibility. Kalscheuer et al. [86] demonstrated potential approaches by injecting human hematopoietic stem cells (HPSCs) aspirated from the bone marrow into the blood of hosts to reconstruct individual donor immune systems; this “personalized immune” mouse models may provide a way to study the human tumor immune system in vivo. More recently, two research groups reported their successful experience in generating humanized NSG (Hu-NSG) mice either by engrafting human fetal liver CD34^+^-purified HPSCs or human peripheral blood mononuclear cells (hPBMCs) into NSG mice and then used them for establishing huNSG-PDX or huNSG-CDX osteosarcoma models (Fig. 3), demonstrating the promising utility as an in vivo model to test drugs targeting the PD-1/PD-L1 axis [87, 88]. More importantly, these two studies disclosed the possibility that human tumors could grow in humanized mice regardless of the mismatched human leukocyte antigen (HLA) status. However, whether this kind of discrepancy between tumor and immunocytes would affect the results of preclinical immunotherapy testing has not been clarified. Dietmar et al. [89] demonstrated that human signal regulatory protein alpha (*SIRPA*) and interleukin 15 (*IL15*) knock-in mouse on a *Rag2*^−*/*−^*Il2rg*^−*/*−^ background could support efficient development of functional maturation of both circulating and tissue-resident human CD8^+^ T lymphocyte subsets and NK cells, thus enabling the investigation of CD8^+^ T and NK cell-based treatments in vivo. The novel gene editing technology CRISPR-Cas9 could also be applied for the development of humanized mice models by substitute part of the mouse gene with a particular gene of human origin to express functional components of the human immune system [90], such as PD-1/PD-L1, thus enabling the testing of some immune-targeting agents. Instead of constructing murine models with intact human immune systems, some studies have reported their success in generating xenograft models by transplanting human tumor cell lines into immunosuppressed or immunotolerant mice to construct xenografts with intact murine immune systems [91, 92]. Using this approach to establish immunocompetent PDX models may be a promising strategy to optimize current immunodeficient models.

PDX models represent a group of promising next generation pre-clinical models, meanwhile, three-dimensional (3D) in vitro tumor models, such as tumor spheroids and tumor organoids, are also emerging as feasible platforms for cancer research. 3D tumor models originate from tumor tissues or single-cell suspensions of tumor cell-lines, patient-derived tumor cells and tumor steam cells are also capable of overcoming the limitations of traditional 2D monolayer cell cultures by accurately reflecting sophisticated cell to extracellular matrix interactions and tumor heterogeneity, however, issues like defining the optimal in vitro culture condition of different tumors, lack of vascularization and immune components in 3D culture systems still need to be addressed in future [93]. When compared to PDXs, the major advantage of 3D tumor models is that they are more inexpensive, faster, and easier to be established [93, 94], while PDXs owns several irreplaceable merits, for example, it allows monitoring tumor neovascularization, distant metastasis, and investigating the side effects of drugs. Interestingly, several research groups have demonstrated that patient-derived organoids (PDOs) and PDXs could be interconverted with high efficiency [95, 96]. In light of this result, we can speculate that PDO may serve as an intermediate states to reduce the time and improve the take rate in generating PDX, while PDX model can act as in vivo model to make up the deficiency of PDO. Making full use of the specific advantages of each model would throw a combination punch to the plight of anti-tumor research.

## Conclusions

It is becoming increasingly clear that PDX models that more closely recapitulate the biological properties of patient tumors are promising substitutes of traditional cell-line xenograft models for developing effective sarcoma therapeutic strategies. For now, PDX models can provide valuable information and chance of cure for future sarcoma patients; however, they seem unlikely to guide real-time clinical decision and improve the prognosis of the original patient. To establish personalized treatment for people with musculoskeletal malignancies through PDX models, consistent efforts for finding proper approaches for enhancing engraftment success rate, reducing the time of PDX model generation, and narrowing down the differences in the tumor microenvironment and heterogeneity between human and PDX models should be further addressed in future studies. Meanwhile, because of the rarity and significant heterogeneity of musculoskeletal malignancies, individual PDX research groups should report their experiment data and methodological information with a standardized criterion to facilitate data integration and resource sharing, which is of paramount importance to identify an optimal method of generating PDX models and reducing unnecessary duplication, thus accelerating the progress of sarcoma research.

## Abbreviations

PDX: patient-derived xenograft
CTC: circulating tumor cell
GNs: granulocytes
DCs: dentric cells
NK: natural killer
NS: NOD-Scid
Scid: severe combined immunodeficiency
NOD: nonobese diabetic
NSG(NOG): NOD.Cg-PrkdcscidIL2rgtm1Sug/Jic or NOD/Shi-scid IL-2Rγnull
STS: soft tissue sarcoma
OS: osteosarcoma
CDS: chondrosarcoma
EWS: Ewing sarcoma
RMS: rhabdomyosarcoma
LPS: liposarcoma
LMS: leiomyosarcoma
SS: synovial sarcoma
SCS: spindle cell sarcoma
HGS: high grade sarcoma
UPS: undifferentiated pleomorphic sarcoma
FBS: fibrosarcoma
AGS: angiosarcoma
MPNST: malignant periphery nerve sheath tumor
NOS: unclassified sarcoma
EPS: epithelioid sarcoma
SFT: solitary fibrous tumor
P: primary tumor
M: metastatic tumor
SC: subcutaneous
OT: orthotopic
SR: surgical resection
Bp: biopsy
Cm: chemotherapy
N: not received
NA: not available
